# Nitric Oxide-Induced Activation of the AMP-Activated Protein Kinase α2 Subunit Attenuates IκB Kinase Activity and Inflammatory Responses in Endothelial Cells

**DOI:** 10.1371/journal.pone.0020848

**Published:** 2011-06-06

**Authors:** Elke Bess, Beate Fisslthaler, Timo Frömel, Ingrid Fleming

**Affiliations:** Centre for Molecular Medicine, Institute for Vascular Signalling, Goethe University, Frankfurt am Main, Germany; Institut National de la Santé et de la Recherche Médicale, France

## Abstract

**Background:**

In endothelial cells, activation of the AMP-activated protein kinase (AMPK) has been linked with anti-inflammatory actions but the events downstream of kinase activation are not well understood. Here, we addressed the effects of AMPK activation/deletion on the activation of NFκB and determined whether the AMPK could contribute to the anti-inflammatory actions of nitric oxide (NO).

**Methodology/Principal Findings:**

Overexpression of a dominant negative AMPKα2 mutant in tumor necrosis factor-α-stimulated human endothelial cells resulted in increased NFκB activity, E-selectin expression and monocyte adhesion. In endothelial cells from AMPKα2^-/-^ mice the interleukin (IL)-1β induced expression of E-selectin was significantly increased. DETA-NO activated the AMPK and attenuated NFκB activation/E-selectin expression, effects not observed in human endothelial cells in the presence of the dominant negative AMPK, or in endothelial cells from AMPKα2^-/-^ mice. Mechanistically, overexpression of constitutively active AMPK decreased the phosphorylation of IκB and p65, indicating a link between AMPK and the IκB kinase (IKK). Indeed, IKK (more specifically residues Ser177 and Ser181) was found to be a direct substrate of AMPKα2 in vitro. The hyper-phosphorylation of the IKK, which is known to result in its inhibition, was also apparent in endothelial cells from AMPKα2^+/+^ versus AMPKα2^-/-^ mice.

**Conclusions:**

These results demonstrate that the IKK is a direct substrate of AMPKα2 and that its phosphorylation on Ser177 and Ser181 results in the inhibition of the kinase and decreased NFκB activation. Moreover, as NO potently activates AMPK in endothelial cells, a portion of the anti-inflammatory effects of NO are mediated by AMPK.

## Introduction

The AMP-activated protein kinase (AMPK) is a member of the Snf1/AMPK family of serine/threonine protein kinases and is an evolutionarily conserved sensor of the cellular energy status. Although the AMPK pathway is traditionally thought of as an intracellular fuel gauge and regulator of metabolism, recent evidence indicates that it may also be important for the maintenance of endothelial function and to redress the disturbed redox balance associated with vascular disease. Certainly, the AMPK can influence a number of signaling cascades that would be expected to result in anti-atherosclerotic effects, such as attenuated free radical generation and the activation of angiogenic factors (for review see [1]).

Although the link between cellular metabolism and AMPK activation has been repeatedly demonstrated in tissues such as skeletal and cardiac muscle [2], the precise role played by the AMPK in endothelial cell remains incompletely understood. Indeed, while there are some situations in which activation of the AMPK is reported to depend on an increase in the ADP/ATP ratio e.g. following cell stimulation with rosiglitazone [3], the activation of AMPK by Ca^2+^-elevating agonists such as bradykinin [4], [5] and thrombin [6] has been attributed to the activity of an upstream activating kinase rather than to changes in AMP levels. There are two different isoforms of the catalytic α AMPK subunit (α1 and α2) that are differentially expressed in different tissues. For example, while the α1 isoform predominates in adipose tissue, skeletal muscle and cardiomyocytes express higher amounts of the AMPKα2 [7]. Interestingly, endothelial cells express both α subunits and different groups report the predominance of different isoforms, a finding that may explain the inconsistent dependence on changes in ADP/ATP for stimulation.

We reported previously that the AMPK can be activated by fluid shear stress as well as by NO in endothelial cells, and that it can affect the expression of endothelial cell proteins including, the hydroxy-methylglutaryl coenzyme A reductase, cytochrome P450 2C8, and angiopoietin 2 [8]–[11]. Also the overexpression of dominant negative AMPKα2 in endothelial cells increases basal and tumor necrosis factor (TNF)-α-stimulated E-selectin expression [10]. While the latter findings imply the involvement of the transcription factor nuclear factor κB (NFκB) and there are reports of an attenuated NFκB activation following AMPK activation in different cell types [12]–[15], the molecular mechanisms involved are not clear. Therefore, the aim of the present study was to address the link between AMPK activation and NFκB inhibition as well as to determine whether or not the activation of the AMPK could at least partially account for the effects of NO on NFκB activity and thus adhesion molecule expression.

## Results

### Effect of NO on the activation of AMPK and NFκB

**Figure 1:**
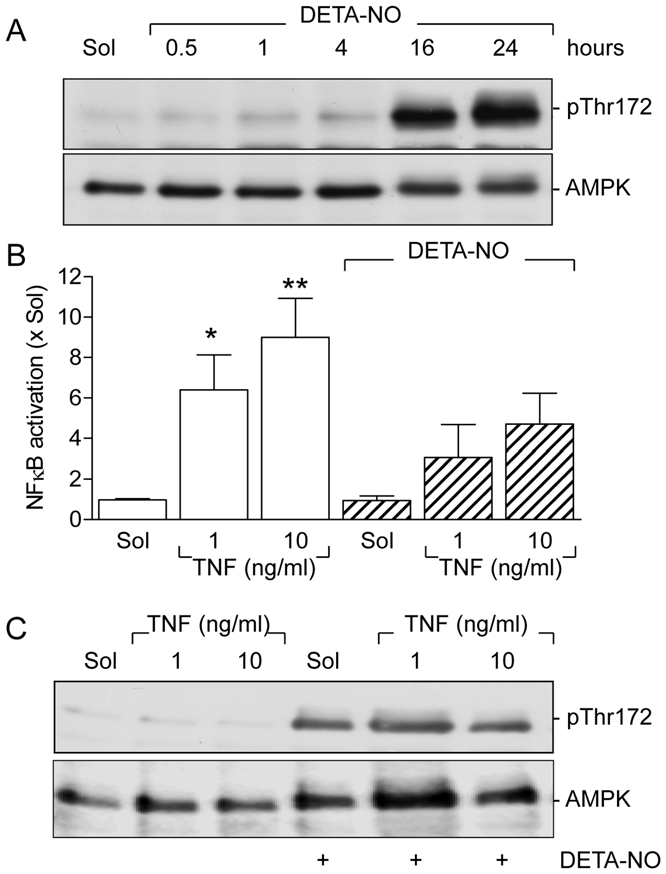
Effect of DETA-NO on AMPK and NFκB activation. Human endothelial cells were treated with either solvent (PBS) or DETA-NO (100 µmol/L) for (**A**) the times indicated or (**C**) 16 hours before stimulation with TNF-α (1 or 10 ng/mL) for 30 minutes. (**A&C**) Western blots showing the consequences of DETA-NO on the phosphorylation of the AMPK (p-Thr172) in the absence (**A**) and presence (**C**) of TNF-α. (**B**) The TNF-α-induced activation of NFκB in the absence and presence of DETA-NO was assessed by EMSA. The bar graph summarizes the results from 4–8 independent experiments; *P<0.05, **P<0.01 versus the appropriate Sol-treated group. The Western blots are representative of 2 additional experiments.

Treatment of primary cultures of human endothelial cells with the NO donor DETA-NO (100 µmol/L) which has a t_½_ of 16 hours, elicited the time-dependent phosphorylation of the AMPK on Thr172 (Figure 1A). The phosphorylation of AMPK by exogenous NO was independent of the donor used as a substance with a markedly faster NO releasing kinetic i.e., DEA-NO, t_½_ 2 minutes, resulted in the more rapid activation of the AMPK i.e., within 2 minutes (Figure S1A). The effects were also concentration-dependent as indicated using a third NO donor with a more delayed NO release (DPTA NO, t_½_ 5 hours; Figure S1B). TNF-α (1 and 10 ng/ml, 30 min) elicited a marked and concentration-dependent increase in NFκB activity (EMSA; Figure 1B), that was attenuated by endothelial cell pre-treatment with DETA-NO (Figure 1B). TNF-α had however no acute effect on the activation of the AMPK in the absence of NO (Figure 1C).

### Effect of constitutively active and dominant negative AMPK mutant on NFκB activation

**Figure 2:**
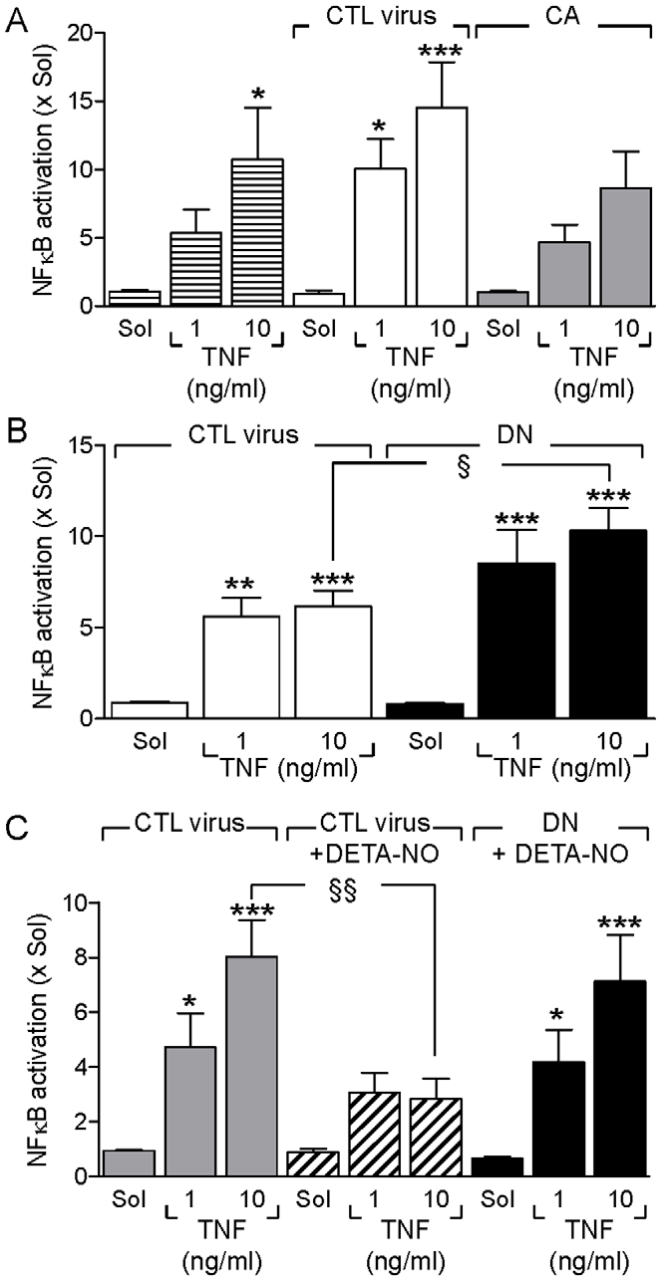
Effect of constitutively active and dominant negative AMPK mutants on NFκB activation. Human endothelial cells infected with either control (CTL) virus or adenoviral constructs encoding (**A**) constitutively active (CA) or (**B&C**) dominant negative (DN) AMPK mutants. After 48 hours cells were treated with TNF-α (1 or 10 ng/mL) for 30 minutes and NFκB activity determined by EMSA. In some experiments (**C**), cells were pre-treated with DETA-NO (100 µmol/L) prior to TNF-α stimulation. The bar graph summarizes the results of data obtained in 4 (A), 6 (B) or 5 (C) independent experiments; *P<0.05, **P<0.01, ***P<0.001 versus the appropriate Sol group and ^§^P<0.05, ^§§^P<0.01, ^§§§^P<0.001 versus cells treated with CTL virus.

To determine the involvement of the AMPK in the prevention of NFκB activation, we assessed the effects of constitutively active and dominant negative AMPKα2 mutants on the TNF-α-induced activation of NFκB. While TNF-α enhanced the activity of NFκB in cells infected with a control virus, this response was blunted in cells overexpressing the constitutively active AMPK (Figure 2A) and potentiated in cells expressing the dominant negative AMPK mutant (Figure 2B). To estimate the influence of the AMPK in the NO-mediated inhibition of TNF-α-induced NFκB activation, cells overexpressing either the control virus or the dominant negative AMPK mutant were exposed to TNF-α in the absence and presence of DETA-NO. As before, pre-treatment with the NO donor attenuated the activation of NFκB (Figure 2C). The effect of NO was however blunted in cells overexpressing the dominant negative AMPK mutant.

### Effect of AMPK deletion on E-selectin and VCAM-1 expression in murine endothelial cells

Theoretically a dominant negative AMPKα2 mutant could affect the activity of the AMPKα1 by, for example, sequestering AMPKβ and γ subunits from the endogenous α1 subunit. To ensure that the effects observed could indeed be attributed to the AMPKα2, we performed additional experiments in endothelial cells from AMPKα2^-/-^ mice or their wild-type littermates (AMPKα2^+/+^).

First, to determine whether or not the loss of one AMPKα subunit could result in the compensatory up regulation of the other we assessed AMPKα1 expression in aortae from α2^+/+^ and α2^-/-^ mice as well as AMPKα2 expression in α1^+/+^ and α1^-/-^ mice. We found that the deletion of the AMPKα2 did not affect α1 expression while the α2 subunit was upregulated in tissue from AMPKα1^-/-^ mice (Figure S2).

**Figure 3:**
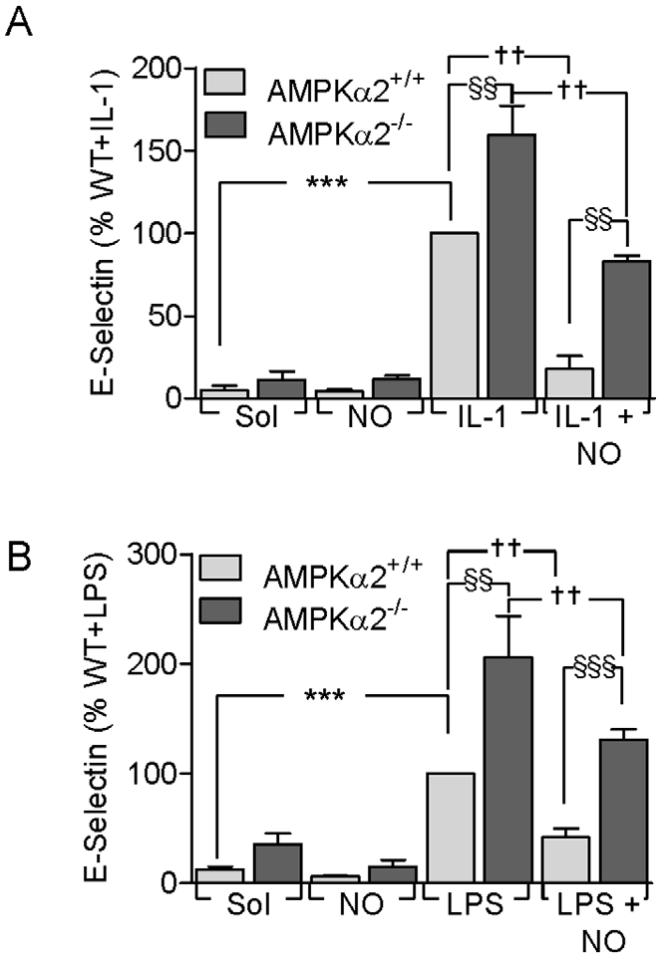
Effect of AMPK deletion on adhesion molecule expression and IκB stability. Lung endothelial cells from AMPKα2^-/-^ mice or their wild-type (+/+) littermates were treated with either IL-1β (10 ng/mL) or LPS (10 ng/mL) in the absence (or presence of DETA-NO (100 µmol/L, 24 hours) for 6 hours. Surface protein expression of (**A**) E-selectin and (**B**) VCAM-1 were determined by FACS analysis. The bar graphs summarize the results of data obtained in 4 independent experiments; ***P<0.001 versus Sol-treated cells, §§P<0.01, §§§P<0.001 and ††P<0.01.

To determine the effect of AMPK deletion on the response to inflammatory mediators, murine microvascular endothelial cells from AMPKα2^+/+^ or AMPKα2^-/-^ mice were exposed to IL-1β (10 ng/mL, 6 hours) or to LPS (10 ng/mL, 6 hours) in the absence and presence of DETA-NO and the surface expression of E-selectin was assessed. The basal expression of E-selectin was slightly (but not significantly) enhanced in cells isolated from the AMPKα2^-/-^ compared to the AMPKα2^+/+^ animals. While the basal expression of the adhesion molecule was attenuated by NO, levels remained slightly elevated in the AMPKα2^-/-^ cells. Cell stimulation with IL-1β (Figure 3A) or LPS (Figure 3B) increased the expression of E-selectin with significantly higher expression levels being detected in AMPKα2^-/-^ cells. TNF-α failed to result in the activation of NFκB in either AMPKα2^+/+^ or AMPKα2^-/-^ cells studied. Pre-treatment of endothelial cells with the NO donor attenuated adhesion molecule expression but again expression was higher (2.5±1.0-fold for IL-1β and 1.7±0.4-fold for LPS) in the cells from AMPKα2^-/-^ mice than in cells from their wild-type littermates.

### Effect of constitutively active and dominant negative AMPK mutants on cell adhesion

**Figure 4:**
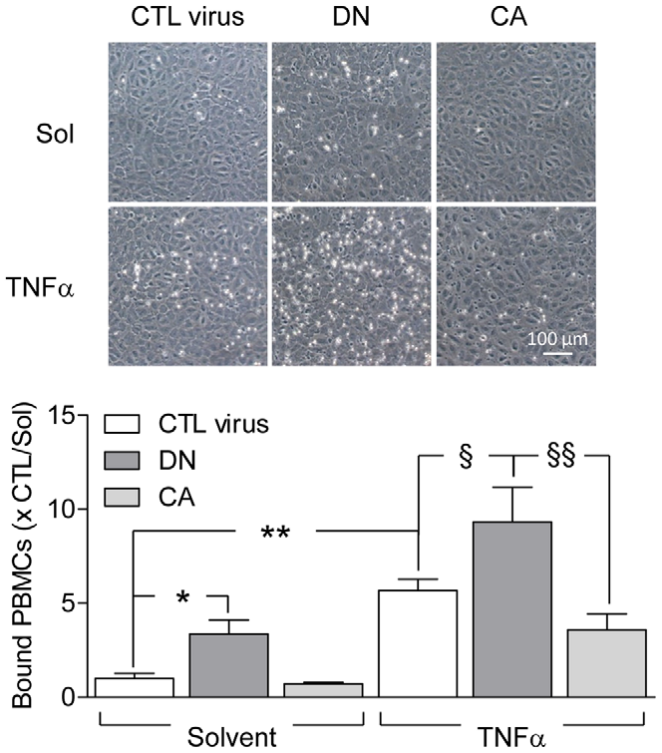
Effect of constitutively active and dominant negative AMPK mutants on monocyte adhesion. Human endothelial cells were infected with either a control (CTL) virus or adenoviral constructs encoding a constitutive active or dominant negative (DN) AMPKα2 mutants. After 48 hours in culture, cells were treated with either solvent or TNF-α (10 ng/ml, 6 hours) before the addition of freshly isolated PBMCs (4×10^5^ per well) for 10 minutes. The bar graph summarizes data obtained in 4 independent experiments, each performed in quadruplicate; *P<0.05, **P<0.01 solvent treated cells expressing CTL virus and §P<0.05, §§P<0.01 versus cells expressing the DN mutant.

Next, we studied the consequences of AMPK mutant overexpression on the adhesion of mononuclear cells to endothelial cells. Consistent with the results obtained using the AMPKα2^-/-^ murine endothelial cells, significantly more mononuclear cells attached firmly to human endothelial cells overexpressing the dominant negative AMPKα2 mutant than those expressing the constitutively active AMPK or treated with the control virus. Cell stimulation with TNF-α (1 ng/mL, 6 hours) resulted in a significant increase in mononuclear cell attachment (Figure 4) that was potentiated in cells expressing the dominant negative, but attenuated in cells expressing the constitutively active AMPK mutants.

### Consequences of AMPK activation/inactivation on IκB

**Figure 5:**
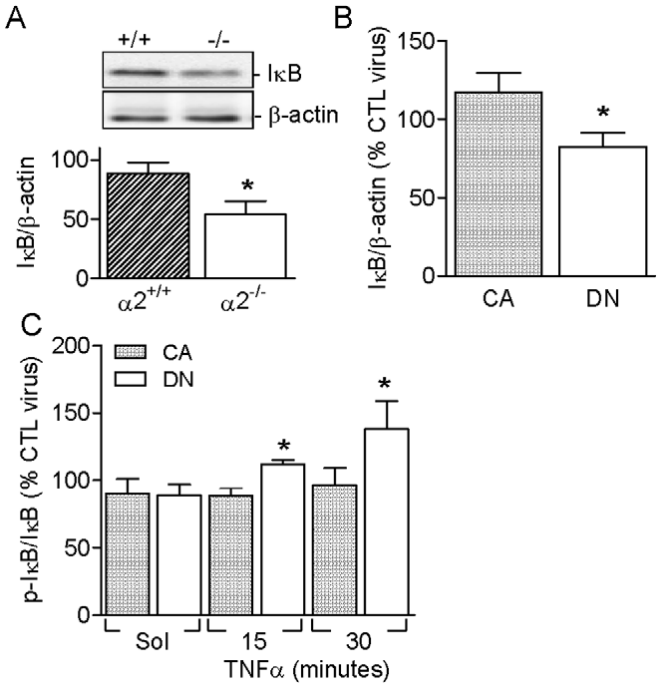
Role of AMPKα2 in regulating the expression and phosphorylation of IκB. Expression of IκB in (**A**) pulmonary endothelial cells from AMPKα2^+/+^ and AMPKα2^-/-^ mice, and (**B**) in COS-7 cells expressing either constitutively active (CA) or dominant negative (DN) AMPKα2. (**C**) IκB phosphorylation in CA- or DN-AMPKα2 expressing COS-7 cells and stimulated with solvent (Sol) or TNF-α (10 ng/mL). Data are expressed relative to values obtained in control virus-infected cells. The bar graphs summarize the results of 4 to 5 independent experiments; *P<0.05 versus CA or ^+/+^.

The activation of NFκB mainly occurs via the phosphorylation and degradation of inhibitory molecules, including IκB. Interestingly, the activation of the AMPK was associated with the increased expression of IκB protein, a phenomenon that was evident on comparing pulmonary endothelial cells from AMPKα2^+/+^ and AMPKα2^-/-^ mice (Figure 5A) or COS-7 cells overexpressing the constitutively active or dominant negative AMPKα2 mutants (Figure 5B). Both these findings support the above evidence indicating that activation of the AMPK results in NFκB inhibition. No difference in IκB expression was detected between AMPKα1^+/+^ and AMPKα1^-/-^ endothelial cells (data not shown).

COS-7 cells expressing constitutively active or dominant negative AMPKα2 were used to determine the consequences of AMPK activation and inhibition on the IκB kinase (IKK)-mediated phosphorylation of IκB independent of potential interference by endogenously generated NO. Neither of the AMPKα2 mutants studied affected the basal IκB phosphorylation. However, following stimulation with TNF-α (10 ng/mL), IκB phosphorylation was enhanced in cells expressing the dominant negative mutant (Figure 5C). The lack of effect of the constitutively active AMPK mutant in these cells can most probably be attributed to the high expression level and activity of the endogenous AMPKα isoforms in this cell line.

The TNF-α-activated IKK complex also phosphorylates p65 on Ser536, a step thought to be required for enhanced p65 transactivation potential and the optimal induction of NFκB target genes [16]. The AMPK also seems to play a role in this pathway as we observed that the expression of the constitutively active AMPK led to a decrease in the TNF-α-stimulated phosphorylation of p65 (Figure S3). Similar results were obtained using human endothelial over expressing the AMPKα2 but not the AMPKα1 subunit (data not shown).

### Consequences of AMPK activation/inactivation on IKK

**Figure 6:**
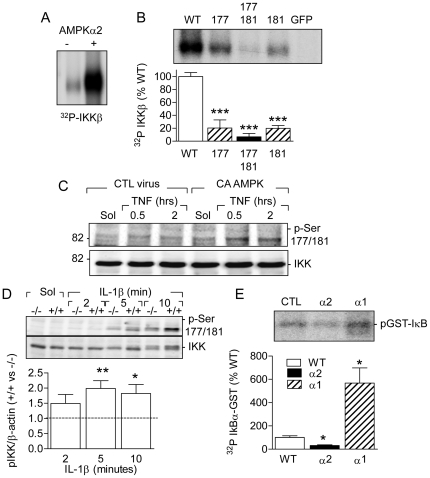
Identification of IKK as an AMPKα2 substrate. (**A**) Autoradiograph showing the in vitro phosphorylation (^32^P) of purified IKKβ by AMPKα2. Similar results were obtained in 2 additional experiments. (**B**) Effect of the substitution of Ser177 and Ser181 with alanine on the AMPKα2-dependent phosphorylation of IKKβ. GFP was used instead of IKKβ as a negative control. (**C**) Phosphorylation of IKK in solvent (Sol)- or TNF-α-stimulated COS-7 cells 48 hours after infection with either CTL or constitutively active (CA) AMPKα2 adenoviruses. Similar results were obtained in 2 additional experiments. (**D**) Phosphorylation of IKK in solvent (Sol)- or IL-1β -stimulated endothelial cells from AMPKα2^+/+^ and α2^-/-^ mice. (**E**) Autoradiograph of phosphorylated GST-IκB after IKK activity assay in the presence of protein A/G (CTL) or immunoprecipitated AMPKα2 or AMPKα1 subunits. Similar results were obtained in three different experiments. The bar graphs summarize the data from 4 (B and E) to 8 (D) independent experiments; *P<0.05, **P<0.01 ***P<0.001 versus WT or ^+/+^.

Given that AMPK activation attenuated the phosphorylation of IκB as well as p65 we proposed that the AMPK is able to attenuate NFκB activation by phosphorylating and inhibiting the IKK. In *in vitro* kinase assays using the purified AMPKα2 (together with β1 and γ1) we identified IKKβ as an AMPK substrate (Figure 6A).

To determine the site(s) in IKKβ phosphorylated by AMPK we replaced its major phosphorylation sites i.e. Ser177 and Ser181 with alanine by site-directed mutagenesis, and overexpressed these mutants in COS-7 cells. The wild-type and mutant IKKβ were then immunoprecipitated and incubated with AMPKα2. We found that the mutation of IKKβ on Ser177 and Ser181 resulted in a lower level of phosphorylation than the wild-type enzyme. Moreover, phosphorylation was barely detectable in the S177A/S181A double mutant. When IKKβ was replaced with GFP, no phosphorylation was observed in the presence of AMPKα2 (Figure 6B). Also in COS-7 cells overexpressing a constitutively active AMPKα2 mutant the phosphorylation of IKK (the antibody used recognizes phospho Ser177 and 181) was increased compared to that detected in cells treated with a control virus (Figure 6C). A similar, approximately 2-fold increase in pIKK levels was observed in murine endothelial cells stimulated with IL-1β (30 ng/mL, Figure 6D) in that the phosphorylation of IKK was consistently greater in cells from AMPKα2^+/+^ versus α2^-/-^ mice. Signals from solvent-treated cells were too low to quantify. There was no difference in the IL-1β -induced phosphorylation in AMPKα1^+/+^ and AMPKα1^-/-^ mice (Figure S4).

To assess IKKβ activity we next determined the phosphorylation of GST-IκBα in the presence of wild-type IKKβ and either AMPKα1 or α2. We found that the phosphorylation of IκBα was reduced in the presence of AMPKα2 but not AMPKα1 (Figure 6E).

## Discussion

**Figure 7:**
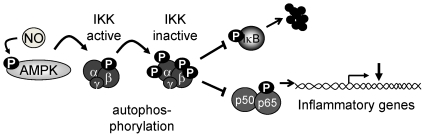
Scheme of the mechanism proposed. NO Initiates the activation of the AMPKα2 in endothelial cells which in turn phosphorylates and activates the β-subunit of the IKK. The latter also induces a higher rate of IKK auto-inactivation and thus attenuates the activation of NFκB and the expression of inflammatory genes.

The results of the present investigation indicate that the AMPKα2 subunit plays an important role in regulating inflammatory responses, adhesion molecule expression (E-selectin and VCAM-1) and monocyte adherence to endothelial cells. These effects could be related, at least partly to the AMPK-mediated phosphorylation of IKK and subsequent inhibition of IκB and p65 phosphorylation as well as DNA binding (see Figure 7). Moreover, three different NO donors were able to activate the AMPK and the NO-mediated inhibition of NFκB activation was attenuated by a dominant negative AMPK in human endothelial cells as well as in endothelial cells from AMPKα2^-/-^ mice. Thus, it appears that the NO-mediated inhibition of NFκB activity is, at least partially, dependent on the activation of the AMPKα2 subunit.

The possibility that the AMPK can regulate the DNA binding activity of NFκB was initially indicated by the fact that overexpression of a dominant negative AMPK increased, while a constitutively active AMPK decreased the TNF-α-induced expression of E-selectin [10]. There have since been reports linking AMPK with NFκB in endothelial cells, but the overall outcome of kinase activation is controversial as both NFκB inhibition [12], [17]–[19] and activation [20]–[23] have been reported. Whether or not this discrepancy can be attributed to the parallel activation/inactivation of associated regulatory mechanisms and signal transduction pathways, remains to be determined. One important consideration is that much of the published data was generated using the AMPK activator, 5-aminoimidazole-4-caboxymide-1-β-D-ribofuranoside (AICAR), which can elicit AMPK-independent effects [24], and substances such as compound C and iodotubercidin which are by no means specific inhibitors of the AMPK. Therefore, one focus of the current investigation was to make use of the available constitutively active and dominant negative AMPK mutants and endothelial cells from AMPKα2^+/+^ and AMPKα2^-/-^ mice to determine which of the catalytic subunits was most likely to affect transcription factor activity. The results obtained using murine lung endothelial cells indicate that the AMPKα2 plays a prominent role in modulating NFκB phosphorylation in endothelial cells.

Which point in the NFκB activation cascade could be affected by AMPK? NFκB is a dimer consisting of the transcription factors p65 (RelA) or p50, and under resting conditions it is associated with its inhibitory protein IκB which retains the complex in the cytosol. The activation of NFκB mainly occurs via the phosphorylation of IκB, which results in its degradation - leaving the NFκB dimer free to translocate into the nucleus and stimulate transcription [16]. Using dominant negative and constitutively active AMPK mutants we found that the phosphorylation of IκB (residue Ser32 or Ser36) was increased when the AMPK was inhibited but decreased when AMPK activity was increased. We also observed the attenuated basal expression of IκB in the AMPKα2^-/-^ versus AMPKα2^+/+^ endothelial cells. As the expression of IκB is known to be regulated by the activity of NFκB [25], these data further highlight the importance of the AMPKα2 subunit in the regulation of transcription factor activity.

The serine phosphorylation of IκB is mediated by a large multi-unit complex containing two catalytic subunits (IKKα and IKKβ) as well as the regulatory subunit IKKγ or NEMO, which has no kinase domain. The p65 subunit is also a IKK substrate and p65 phosphorylation on Ser536 is thought to be required for enhanced p65 transactivation potential and the optimal induction of NFκB target genes [16]. Given that we observed that both AMPK activation and a constitutively active AMPK mutant decreased the phosphorylation of IκB and p65, it seemed logical to conclude that the AMPK is able to modulate the activity of the IKK complex.

To date there has only been circumstantial evidence to suggest a role of the AMPK in the modulation of IKK activity. For example, AICAR has been reported to be without effect on IKK in rat skeletal muscle cells [26] but to attenuate IκB phosphorylation in IL-18-stimulated cardiac microvascular endothelial cells [27]. Moreover, the overexpression of a dominant-negative AMPK in TNF-α–stimulated mouse macrophages resulted in the accelerated and exaggerated degradation of IκB [28]. Similarly, during the preparation of this manuscript the loss of AMPK activity was reported to result in increased IκBα degradation in cultured endothelial cells [23], a finding we have confirmed in native endothelial cells. The results of the present study clearly indicate that the AMPK can directly phosphorylate and attenuate the activity of the IKK in native and cultured endothelial cells. In *in vitro* assays we identified IKKβ as an AMPK substrate and were able to detect the elevated phosphorylation of IKK in intact AMPK-overexpressing cells. The phosphorylation of IKKβ on Ser 177 and/or 181 causes the rapid phosphorylation of a C-terminal serine cluster which in turn elicits the auto-inhibition of kinase activity and is thought to be an effective means of limiting the duration of IKK activation [29]. Our data suggest that the AMPKα2 is able to phosphorylate both of these serine residues as mutation of a single site reduced phosphorylation which was only barely detectable in a double S177A/S181A mutant. That the AMPK-mediated phosphorylation of the IKK most likely results in NFκB inhibition was demonstrated by the fact that the IKK-dependent phosphorylation of a IκB-GST construct was markedly attenuated in the presence of the AMPKα2 but not the AMPKα1 subunit. Moreover, the expression of IκB was significantly higher in human endothelial cells overexpressing the constitutively active AMPKα2 and in AMPKα2^+/+^ versus AMPKα2^-/-^ mouse endothelial cells.

The AMPK has been implicated in the phosphorylation and activation of the endothelial NO synthase (eNOS), at least *in vitro*, and thus one further mechanism by which the AMPK could affect NFκB would be by modulating basal NO output (reviewed in [1]); which is known to decrease NFκB activity [30]. However, using COS-7 cells which do not express endogenous eNOS, we found that AMPK activation and the constitutively active AMPK were sufficient to attenuate the TNF-α-induced phosphorylation of IκB and p65. Thus in our hands, the AMPK-dependent inhibition of NFκB was not dependent on NO production. In fact, we were able to confirm that NO donors are effective activators of the AMPK a finding that fits well with previous reports in endothelial cells that AMPK activation is markedly attenuated in the presence of a NOS inhibitor or in cells from eNOS^-/-^ mice [5], [9]. How NO activates the AMPK remains to be elucidated but may well be related to the activation of the Ca^2+^/calmodulin-dependent protein kinase kinase β, a well known AMPK kinase [5].

Taken together, the results of the present investigation indicate that the activation of the AMPK in endothelial cells can limit inflammatory responses via the phosphorylation and inhibition of IKK activity. Moreover, as the AMPK could be activated by NO, the AMPK dependent inhibition of IKK activity may contribute to the anti-inflammatory and anti-atherosclerotic actions of the endothelium-derived autacoid.

## Materials and Methods

### Materials

Antibodies for Western blotting directed against phospho-Thr172 AMPK and total AMPK, phospho-Ser536 NFκB, phospho-Ser32/36 IκB and phospho Ser 177/181 IκB kinase (IKK) were purchased from New England Biolabs (Frankfurt, Germany). Antibodies recognizing IκB, NFκB or IKK from Santa Cruz Biotechnology (Heidelberg, Germany). Horseradish peroxidase conjugated secondary antibodies were from Calbiochem (Merck, Darmstadt, Germany) and the fluorescent antibodies recognizing E-selectin and VCAM-1 used for FACS analyses were from BD Biosciences (Pharmigen, Germany). TNF-α and interleukin (IL)-1β, were from PeproTech (Cell Concept, Umkirch, Germany), and DETA-NONOate (DETA-NO), DEA-NONOate and DPTA-NONOate were from Alexis (Lörrach, Germany). The antibody against β-actin and other chemicals were obtained from Sigma (Munich, Germany).

### Cell culture

Human umbilical vein endothelial cells were isolated and cultured as described [31]. The use of human material in this study conforms to the principles outlined in the Declaration of Helsinki [32] and was confirmed in written form by the members of the Ethic-Commission of the Medical Faculty of the Goethe-University (Frankfurt am Main, Germany) and the donors gave verbal consent. Murine lung endothelial cells were isolated and cultured as described [8], from AMPKα1^-/-^ or AMPKα2^-/-^ mice or the respective littermate wild-type animals [33], [34] (kindly provided by Benoit Viollet, Paris via the European Mouse Mutant Archive, Munich, Germany). The investigation conforms with the Guide for the Care and Use of Laboratory Animals published by the European Commission Directive 86/609/EEC. Both the University Animal Care Committee and the Federal Authority for Animal Research at the Regierungspräsidium Darmstadt (Hessen, Germany) gave written approval to the study protocol (# F28/17). For the isolation of the pulmonary endothelial cells, mice were sacrificed using 4% isoflurane in air and subsequent exsanguination. COS-7 cells were purchased from the American Tissue Culture Collection (LCG Standards, Wesel, Germany) and cultured in Minimal Essential Medium (Invitrogen, Karlsruhe, Germany) supplemented with 8% fetal calf serum, pyruvate, non essential amino acids and gentamycin.

### Adenoviral transduction of endothelial cells

Subconfluent endothelial cells (1^st^ passage) were infected with adenoviruses (provided by Ken Walsh, Boston and Benoit Viollet, Paris, France) to over-express constitutively active AMPKα2 [35], or dominant-negative AMPKα2 [36], as described [10]. As the viral backbones of the constitutive active and dominant negative viruses were not identical, the observed effects were always analyzed to the respective control virus.

### Immunoblotting

Protein samples were heated with SDS-PAGE sample buffer and separated by SDS-PAGE as described [8]. Proteins were detected using their respective antibodies and enhanced chemiluminescence using a commercially available kit (Amersham, Germany). To assess the phosphorylation of proteins, either equal amounts of protein from each sample were loaded twice and one membrane incubated with the phospho-specific antibody and the other with an antibody recognizing total protein, or blots were re-probed with the appropriate antibody.

### Electrophoretic Mobility Shift Assay (EMSA)

Nuclear and cytosolic proteins were isolated, and binding to γ[^32^P]-ATP (Hartmann Analytic, Braunschweig, Germany)-labeled double-stranded oligonucleotides containing the consensus sequence of the binding site for transcription factor NFκB (5-AGT TGA GGG GAC TTT CCC AGG C-3, Santa Cruz) was assessed as described [37].

### Flow cytometry

Following stimulation endothelial cells were harvested using accutase (PAA laboratories, Coelbe, Germany) and washed twice with PBS. After blocking (1% BSA, 15 minutes, 4°C) cells were incubated with the specific conjugated antibodies (30 minutes, 4°C), washed twice with PBS and fixed in 1% paraformaldehyde. Antibody binding was analyzed using a flow cytometer (FACSCalibur, BD) by counting 20,000 cells per sample. The values are presented following subtraction of the isotype-matched, control IgG.

### Adhesion assay

Peripheral blood mononuclear cells were freshly isolated from blood obtained from healthy volunteers using a Biocoll® (1.077 g/ml) gradient (Biochrom AG, Berlin, Germany) according to the manufacturer's protocol. Confluent cultures of human endothelial cells were stimulated with TNF-α for 6 hours, and were washed with culture medium before freshly isolated mononuclear cells (400,000 per 12 well) were added. After 10 minutes of incubation the non-adherent cells were removed the number of firmly adherent cells was quantified.

### IKKβ kinase assays

The phosphorylation of IKKβ by AMPKα2 and the activity of IKK β were assessed in in vitro kinase assays. Ser177 and Ser181 in a flag-tagged IKK plasmid [38] (Addgene, Cambridge, MA) were mutated to alanine by site-directed mutagenesis (Quick exchange Kit, Agilent, Böblingen, Germany) using specific oligonucleotides (Biospring, Frankfurt, Germany). COS-7 cells were transfected with a plasmid encoding either the wild-type flag-tagged IKKβ or one of the S177A, S181A or S177A/181A mutants, which were then immunoprecipitated using a Flag antibody (Invitrogen, Karlsruhe, Germany). The AMPKα2 subunit was also immunoprecipitated from COS-7 cells and the in vitro kinase reaction performed as described [9]. In some experiments the activity of the IKKβ was detected by monitoring the phosphorylation of a GST-IκB fusion protein (kindly provided by Fumiyo Ikeda, Frankfurt).

### Statistical analysis

Values are expressed as the mean ± SEM and statistical evaluation was performed using Student's *t* test for unpaired data and one-way ANOVA or ANOVA for repeated measures followed by followed by a Bonferroni *t* test where appropriate. Values of *P*<0.05 were considered statistically significant.

## Supporting Information

Figure S1 **Effect of NO donors on the phosphorylation of AMPK.** (**A**) Human endothelial cells (passage 2) were treated with either solvent (Sol) or DEA-NO (100 µmol/L, t½ 16 minutes) for up to 30 minutes. Identical results were obtained in two additional experiments. (**B**) Human endothelial cells were treated with different concentrations of the NO donor DPTA NONOate (t½ 5 hours) and the phosphorylation of the AMPK was detected by Western blotting. The graph summarizes the data obtained in 3 independent experiments; **P<0.01 versus the appropriate Sol-treated group.
(TIF)

Figure S2 **Effect of AMPKα subunit deletion on the expression of the second isoform in aortic lysates.** While deletion of the AMPKα2 subunit had no effect on the expression of the AMPKα1 isoform, the deletion of AMPKα1 induced a compensatory increase in AMPKα2 expression. Each lane represents tissue from a different animal and identical results were obtained in tissue from 4 additional animals.
(TIF)

Figure S3 **Role of AMPKα2 in regulating the expression and phosphorylation of p65.** The phosphorylation of p65 NF-kB was assessed in COS-7 cells expressing either constitutively active (CA) or dominant negative (DN) AMPKα2 and stimulated with solvent (Sol) or TNF-α (10 ng/mL). Data are expressed relative to values obtained in control virus-infected cells. The bar graph summarizes the results of 4 to 5 independent experiments; *P<0.05 versus CA.
(TIF)

Figure S4 Effect of the AMPKa1 deletion on the IL1β (30 ng/mL)-mediated phosphorylation of IKKα/β and IκB in mouse lung endothelial cells. The bar graph summarizes the results of 4–5 independent experiments.
(TIF)
