# Supplementary figures and images for: Nitric Oxide-Induced Activation of the AMP-Activated Protein Kinase α2 Subunit Attenuates IκB Kinase Activity and Inflammatory Responses in Endothelial Cells
Source: PLoS One. 2011 Jun 6;6(6):e20848. doi: 10.1371/journal.pone.0020848 (PMC3108981; doi:10.1371/journal.pone.0020848)

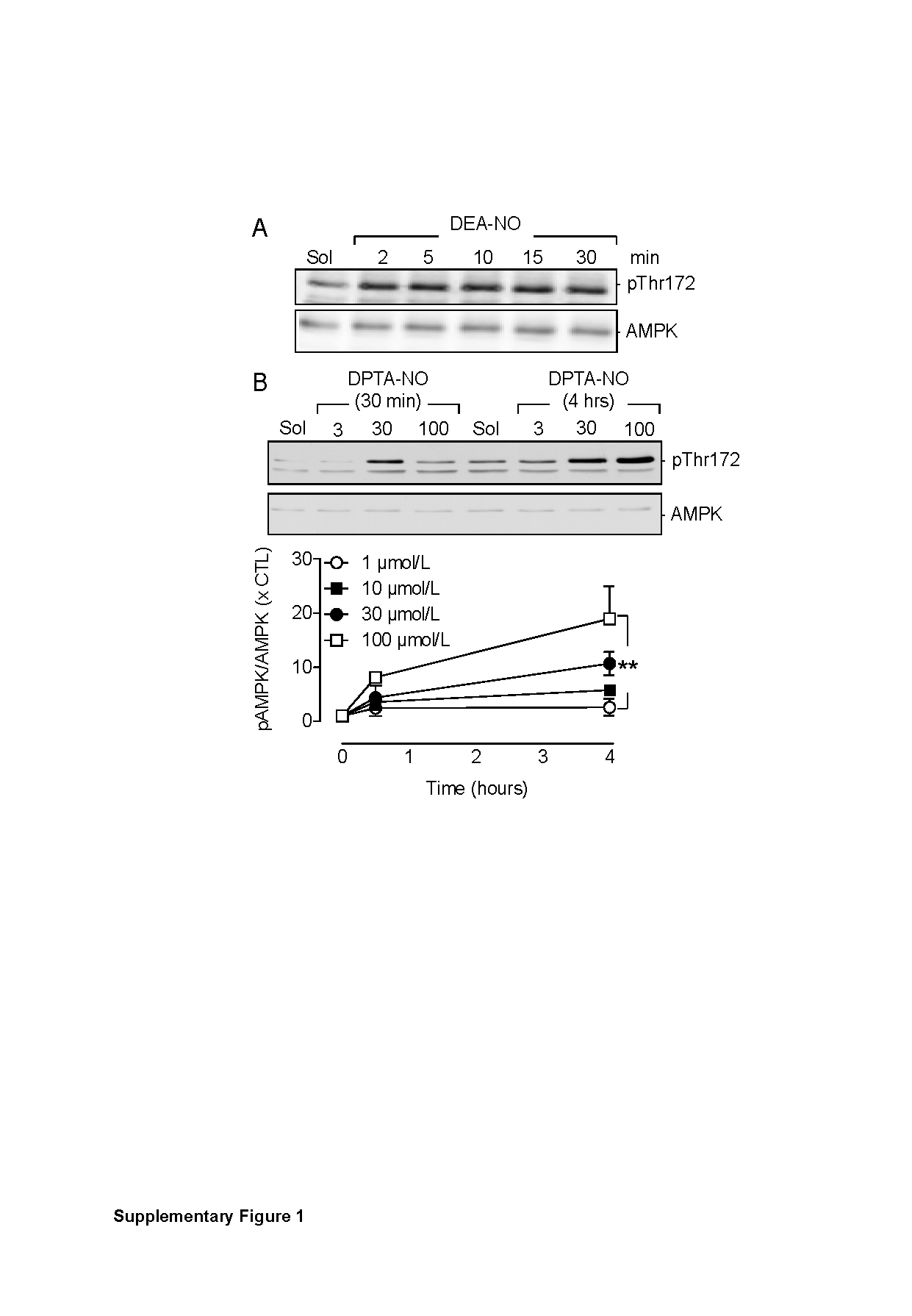

Supplement: Figure S1 — Effect of NO donors on the phosphorylation of AMPK. (A) Human endothelial cells (passage 2) were treated with either solvent (Sol) or DEA-NO (100 µmol/L, t½ 16 minutes) for up to 30 minutes. Identical results were obtained in two additional experiments. (B) Human endothelial cells were treated with different concentrations of the NO donor DPTA NONOate (t½ 5 hours) and the phosphorylation of the AMPK was detected by Western blotting. The graph summarizes the data obtained in 3 independent experiments; **P<0.01 versus the appropriate Sol-treated group. (TIF) [file pone.0020848.s001.tif]

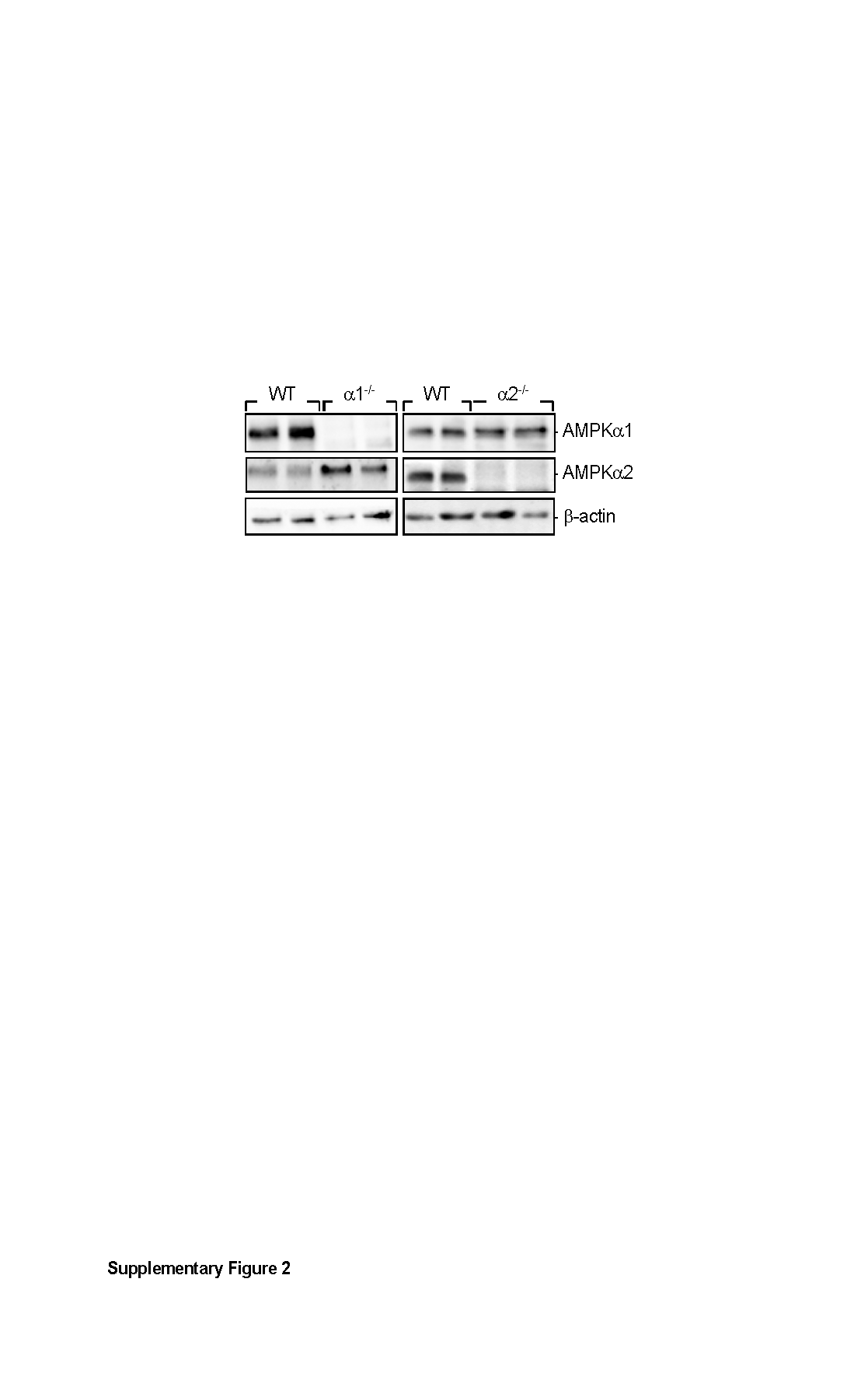

Supplement: Figure S2 — Effect of AMPKα subunit deletion on the expression of the second isoform in aortic lysates. While deletion of the AMPKα2 subunit had no effect on the expression of the AMPKα1 isoform, the deletion of AMPKα1 induced a compensatory increase in AMPKα2 expression. Each lane represents tissue from a different animal and identical results were obtained in tissue from 4 additional animals. (TIF) [file pone.0020848.s002.tif]

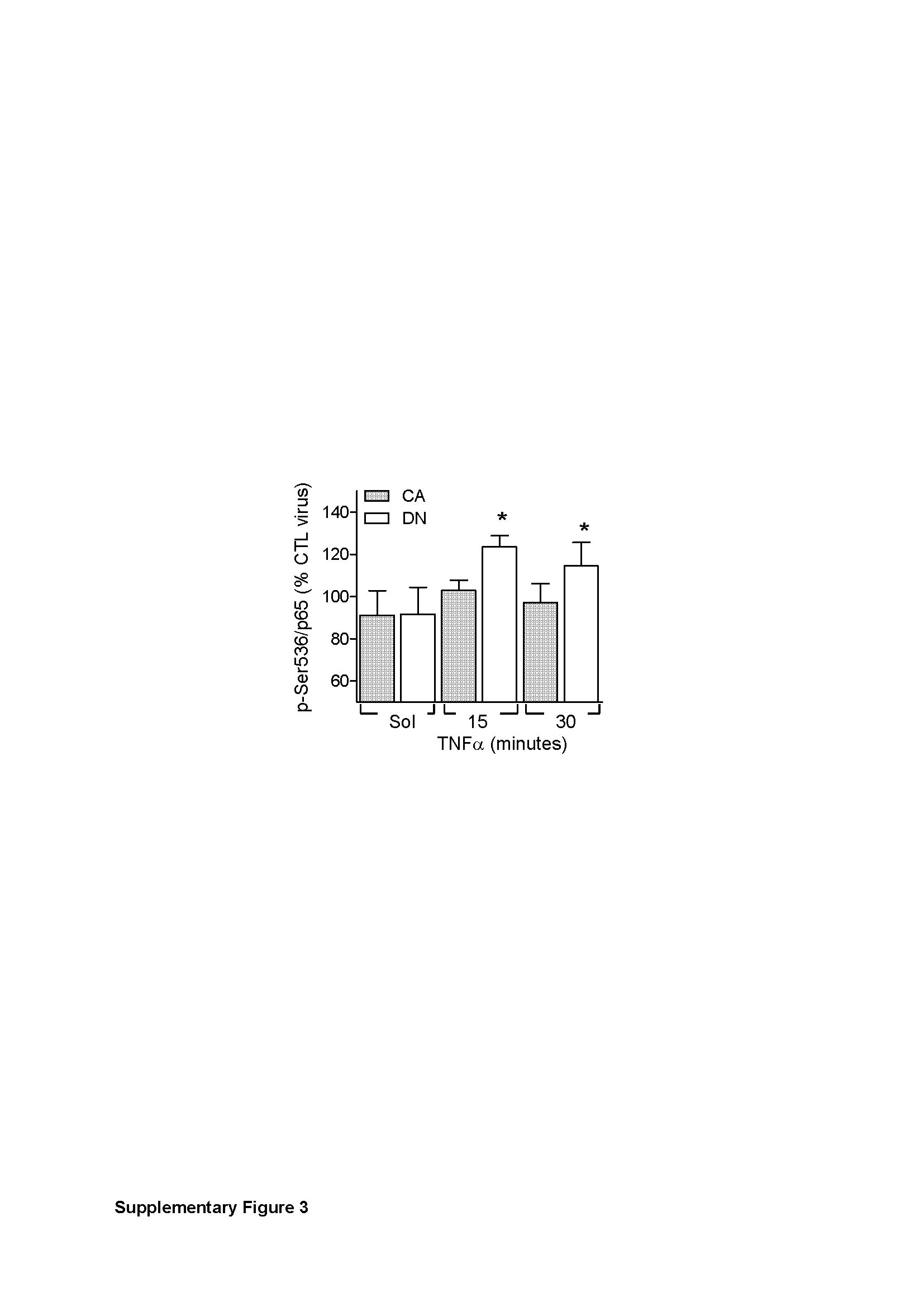

Supplement: Figure S3 — Role of AMPKα2 in regulating the expression and phosphorylation of p65. The phosphorylation of p65 NF-kB was assessed in COS-7 cells expressing either constitutively active (CA) or dominant negative (DN) AMPKα2 and stimulated with solvent (Sol) or TNF-α (10 ng/mL). Data are expressed relative to values obtained in control virus-infected cells. The bar graph summarizes the results of 4 to 5 independent experiments; *P<0.05 versus CA. (TIF) [file pone.0020848.s003.tif]

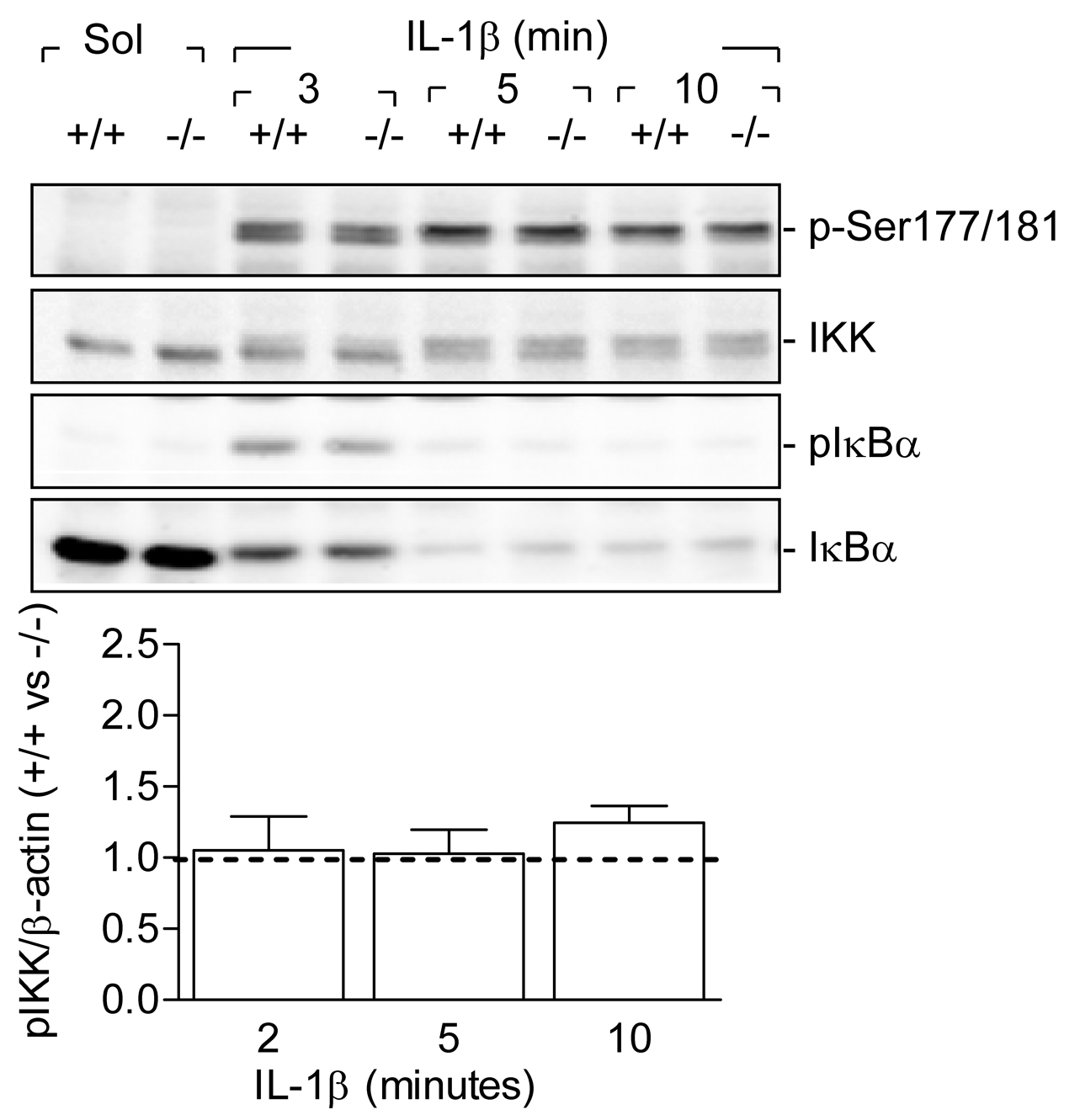

Supplement: Figure S4 — Effect of the AMPKa1 deletion on the IL1β (30 ng/mL)-mediated phosphorylation of IKKα/β and IκB in mouse lung endothelial cells. The bar graph summarizes the results of 4–5 independent experiments. (TIF) [file pone.0020848.s004.tif]
